# Effect of a walnut meal on postprandial oxidative stress and antioxidants in healthy individuals

**DOI:** 10.1186/1475-2891-13-4

**Published:** 2014-01-10

**Authors:** Ella H Haddad, Natasha Gaban-Chong, Keiji Oda, Joan Sabaté

**Affiliations:** 1 Department of Nutrition, School of Public Health, Loma Linda University, Loma Linda, CA 92350, USA; 2 Nutritional Services, Loma Linda University Medical Center, Loma Linda, CA 92350, USA; 3 Department of Biostatistics and Epidemiology, School of Public Health, Loma Linda University, Loma Linda, CA 92350, USA; 4 Department of Nutrition, Loma Linda University, Loma Linda, CA 92350, USA

**Keywords:** Walnuts, Oxidative stress, γ-tocopherol, Catechins, Urolithin A

## Abstract

**Background:**

*In vitro* studies rank walnuts (*Juglans regia*) among the plant foods high in antioxidant capacity, but whether the active constituents of walnuts are bioavailable to humans remains to be determined. The intention of this study was to examine the acute effects of consuming walnuts compared to refined fat on meal induced oxidative stress. At issue is whether the ellagitannins and tocopherols in walnuts are bioavailable and provide postprandial antioxidant protection.

**Methods:**

A randomized, crossover, and controlled-feeding study was conducted to evaluate a walnut test meal compared to one composed of refined ingredients on postprandial serum antioxidants and biomarkers of oxidative status in healthy adults (n = 16) with at least 1 week between testing sessions. Following consumption of a low phenolic diet for one day and an overnight fast, blood was sampled prior to the test meals and at intervals up to 24 hours post ingestion and analyzed for total phenols, malondiadehyde (MDA), oxidized LDL, ferric reducing antioxidant power (FRAP), hydrophilic and lipophilic oxygen radical absorbance capacity (ORAC), uric acid, catechins and urinary excretion of phenylacetate metabolites and of urolithin A.

**Results:**

Mixed linear models demonstrated a diet effect (*P* < 0.001) for plasma γ-tocopherol but not for α-tocopherol with the walnut meal. Following the walnut test meal, the incremental 5 hour area under the curve (AUC_0-5h_) was reduced 7.4% for MDA, increased 7.5% for hydrophilic and 8.5% for lipophilic ORAC and comparable for total phenols, FRAP and uric acid. Oxidized LDL was reduced at 2 hours after the walnut meal. Plasma concentrations of gallocatechin gallate (GCG), epicatechin gallate (ECG) and epicallocatechin gallate (EGCG) increased significantly at 1 hour after the walnut test meal. Quantities of urolithin-A excreted in the urine were significantly higher following the walnut meal.

**Conclusions:**

Compared to the refined control meal, the walnut meal acutely increased postprandial γ-tocopherol and catechins and attenuated some measures of oxidative stress.

## Background

Walnuts (*Juglans regia* L,) originated in central Asia and the Mediterranean region and are one of the oldest tree foods consumed by humans [1]. Epidemiological studies report a dose-dependent negative association between consumption of tree nuts and reduced risk diet-related disorders such as obesity and cardiovascular events [2]. Whereas this benefit is usually attributed to the unsaturated fatty acid composition of nuts, there may be other relevent mechanisms involved including the modulating effect of bioactive nut components on oxidative damage.

Walnuts are rich in the polyunsaturated fatty acids linoleic and α-linolenic at 52.4% and 12.5% of kcals respectively [3] and thus potentially susceptible to oxidation. These lipids are naturally protected by tocopherols in the seed and phenolic compounds in the pellicle or seed coat [4-6]. Walnut phenolics are mostly of the non-flavonoid type belonging to the ellagitannin, or hydolyzable tannins, category [4-15]. Data on walnut phenolic composition is fragmentary and varies with methods used for extraction and analysis (See Additional file 1: Table S1). The levels of polyphenols in the nut are also influenced by genetic factors and environmental growing conditions. It is estimated that aglycone and glycosylated ellagic acid accounts for 64-75% of total phenols in walnuts [14]. Whereas Harnly et al. [16] found no flavan-3-ol monomers in walnuts, Gu and Kelm [9] and Gomez-Caravaca et al. [14] detected small quantities of catechins and proanthocyanidin monomers.

As rich sources of phenolics, walnut extracts have been tested in vitro assay systems such as the ferric reducing antioxidant power (FRAP) and the oxygen radical antioxidant activity (ORAC) and shown to exhibit substantial antioxidant activity [17-20]. An aqueous ethanol extract of walnut kernels showed superoxide dismutase type activity, radical scavenging effect against diphenylpicrylhydrazyl (DPPH) [8], and inhibition of azobisaminopropane dihydrochloride (AAPH) induced LDL oxidation [21]. The acute consumption of walnuts has been associated with postmeal enhancement of antioxidant capacity [22], increased plasma FRAP activity [23], and inhibited inflammatory responses [24]. However, the bioavailability and role of walnut phenolics in these effects has not been fully determined. Polymeric ellagitannins and their hydrolysis product ellagic acid are poorly absorbed as such, but are metabolized by human colonic microflora to yield the more absorbable urolithins and related compounds [25].

In healthy individuals higher fat meals when compared to high carbohydrate meals generally attenuate postprandial lipemia a condition associated with increased oxidative stress and cardiovascular risk [26]. Not known is whether the tocopherols and polyphenols in a walnut meal are bioavailable and consequently provide a measure of antioxidant protection. The purpose of this study is to extend our previous work relating nut intake with antioxidant measures [27] and explore changes in plasma and urine concentrations of tocopherols and phenolic compounds following consumption of a single large dose of walnuts (90 g) compared to that of a refined high-fat test meal. The current study examined postprandial alterations in blood tocopherols, catechins, *in vivo* oxidant markers (MDA, LDL oxidation), measures of antioxidant activity (FRAP, ORAC and uric acid) and urinary metabolites associated with walnut ingestion.

## Methods

### Study design

Sixteen young adults (6 males, 10 females) with a median age 26 y (age range 23 – 44 y) and mean BMI of 22.7 ± 3.4 kg/m^2^ were recruited for the study. Subjects were apparently healthy and nonsmokers as indicated by a general medical questionnaire. They were not taking any medications or nutritional supplements, not pregnant or lactating, not allergic or sensitive to walnuts, and not habitual consumers of tea or coffee at ≤ 2 cups per week of coffee and/or tea. The participants were informed of the purpose and risks of the study, and written informed consent was obtained. The study protocol was approved by the Institutional Review Board of Loma Linda University.

The study followed a single-blind (ie, researchers were blinded) randomized, controlled crossover design with 1 to 4 week separation between testing sessions. The test meals were either a walnut, or control breakfast meal in random order. Participants were provided a polyphenol-free diet (no fruits, vegetables, coffee, tea, chocolate, juice, wine, etc.) on the day prior to the intervention. The meals were energy adjusted for each participant and standardized to provide 50%, 20% and 30% of kcals as carbohydrate, protein and fat respectively. All meals were prepared by the researchers and served in the Nutrition Research Kitchen of the University.

The walnut meal was composed of 90 g of raw shelled walnut kernels and 250 ml of distilled water. The macronutrient composition of the control meal was similar to that of the walnuts meal, but was comprised of refined olive oil, white bread and dried egg-white powder blended with 250 ml of distilled water to make a smoothie. The nutrient composition of the test meals is shown in Table 1.

**Table 1:** **Composition of test meals**^**1**^

	**Control**	**Walnut (90 g)**
Energy, kj	2600	2640
Total fat, g	58.2	58.7
Saturated fat, g	8.03	5.51
Monounsaturated fat, g	42.46	8.04
Polyunsaturated fat, g	6.12	42.46
Protein, g	13.3	13.7
Carbohydrate, g	11.2	12.3
Alpha-tocopherol, mg	8.4	0.63
Gamma-tocopherol, mg	0.5	18.7
Proanthocyanidins, mg	-	60.6^2^
Flavan-3-ol monomers (catechins), mg	-	6.24^2^
Total phenolics, mg GAE	11.8	1400^3^

^1^Nutrient composition values were obtained from USDA Database for Standard Reference unless otherwise specified [3].

^2^Gu et al. [9].

^3^Wu et al. [12].

On the day of testing, after a 12 hour overnight fast, baseline blood samples were drawn at approximately 7:00 am after which participants consumed the test meal. The time at which the ingestion of the test meal began was recorded and participants were allowed 20 min to complete the test meal. Additional blood samples were obtained at 1, 2, 3, and 5 hours following the initiation of the test meal. On the intervention day, food and drinks were restricted to water and a standardized low-phenol meals for lunch and supper. At around 12:00 pm, participants consumed a low-phenol lunch and an additional blood sample was drawn at 8 hours after the test meal. Participants consumed a low-phenol supper at around 6:00 pm, and following an overnight fast, an additional blood sample was obtained the following morning at 7:00 am. For each draw, blood was centrifuged and portions of serum and plasma were stored at -80°C for subsequent analysis. Following administration of the test breakfasts, participants self collected 24-hour urine samples, in 12-hour split collection times. Participants kept urine samples refrigerated until delivery to the laboratory for volume measurement and storage at -80°C.

### Assay methods

#### Serum lipids and uric acid

Serum cholesterol, triacylglycerols and uric acid were determined with reagents, controls, and calibrators from Pointe Scientific, Inc. (Canton, MI) and were assayed on a Bio-tek Synergy HT (Winooski, VT) plate reader.

#### Measures of antioxidant capacity

Antioxidant capacity of plasma was determined by the FRAP assay in a redox-linked colorimetric reaction using methods modified from Benzie and Strain [28]. Plasma was incubated at room temperature with the FRAP reagent and absorbance recorded on a Synergy Analyzer (Bio Tek Instruments) at a wavelength of 593 for 4 minutes. Trolox was used as standard and the results are expressed mmol Trolox equivalents (TE) per liter.

Plasma samples were analyzed for hydrophilic and lipophilic ORAC according to published methods [29,30]. The assay was carried on 48-well microplates using the FLX 800 fluorescent reader (Bio Tek Instruments) with an excitation wave-length of 546 nm and an emission wavelength of 565 nm and the reaction was monitored for 1 hour and 15 minutes. Calculations were made using Microsoft Excel and the data expressed as mmol TE per liter.

#### Measures of lipid oxidation

Plasma MDA was quantified using a method based on formation thiobarbituric acid reactive substances using reverse phase HPLC separation and fluorescence detection as developed by Templar et al. [31]. HPLC analyses were performed with a Shimadzu RF-353 fluorescence detector, LC-10AT pump, and SIL-10 AD auto sampler. Oxidized LDL concentrations on serum were determined by an enzyme-linked immunoassay kit (ALPCO Diagnostics) which specifically tests for MDA-modified LDL.

#### Total phenols, tocopherols and catechins

Total phenolic content of plasma was measured using the Folin-Ciocalteau colorimetric assay on deproteinated samples as described by Serafini et al. [32]. Data are expressed as mmol gallic acid equivalent (GAE) per liter. Plasma tocopherols were determined by normal phase HPLC according to the method of Kramer et al. [33] which uniquely separates γ- from β- tocopherol.

Measurement of plasma catechins samples obtained from 6 randomly selected participants was contracted to Brunswick Laboratories Inc. (Southborough, MA). Following enzymatic hydrolysis of plasma by β-glucuroniase and sulfatase, catechin concentrations were quantified using HPLC with a coulochem electrode array detection according to the method of Lee et al. [34]. Due to limited biospecimen availability, the catechin assays were carried out on fasting samples and at 1 and 2 hours following the test meal.

#### Urine metabolites

Prior to HPLC analysis for urolithin-A, urine samples were enzymatically treated with β-glucoronidase and sulfatase and extracted with diethyl ether as described by Cerda et al. [35]. Urolithin A (95% purity) (3,8-dihydroxy-6*H*-dibenzo[*b,d*]pyran-6-one) standard was obtained from Kylolab S.A. (Murcia,Spain). HPLC separation of the metabolite was carried out on a Symmetry C-18, column (Waters Corp) as described by Seeram et al. [36]. Urine phenolic metabolites on 6 randomly selected participants were measured at the Henning laboratory at the Center for Human Nutrition, University of California according to published protocols [37,38].

#### Statistical analysis

For comparisons of serum lipids and measures of antioxidant status at baseline and 24 hours after the test meal, paired t-tests were used. Normality was confimed by visual inspection of histograms and by the Kolmogorov-Smirnov test. Results were presented as means ± SEM by test meal. For comparisons of biomarker profiles over time between test meals, the incremental 5 hour area under the curve (AUC_0-5h_) was calculated using the trapezoidal rule with the premeal baseline value as the line of reference. The mixed model approach was then used to test the effect of treatment, adjusting for period effect, and treating subjects nexted within sequence as random effects. For plasma catechins, mixed models included treatment, time and their interaction, while adjusting for period and subjects as random effects. The Tukey-Kramer multiple comparison was used to test difference in catechin concentration between test meals at each time point. For mixed model analyses, residual plots were used to check model assumptions. Due to non-normality of data, oxidized LDL values were log-transformed. Results of the analysis were presented as least suqares mean (estimated from the mixed model) and its 95% confidence interval, as well as *P* value comparisons between test meals. Differences were considered significant at *P* < 0.05. All statistical analyses were perfomed using SAS version 9.2 (SAS Institute, Cary, NC).

## Results

Fasting plasma concentrations of lipids and measures of antioxidant status prior to consuming (0 hours) and 24 hours following the test meal are shown in Table 2. No differences were observed in baseline values between test days for any of the measures. In addition, most antioxidant responses showed no differences between fasting concentrations at 24 hours compared to baseline except for FRAP and γ-tocopherol which were higher and triacylglycerols which were lower at 24 hours following the walnut intervention.

**Table 2:** **Fasting circulating lipids and indicators of antioxidant status prior to (0 hour) and 24 hours following consumption of control and walnut meals**^**1**^

	**0-hour**		**24-hour**	
**Measurement**	**Control (n = 16)**	**Walnut (n = 16)**	**Control (n = 16)**	**Walnut (n = 16)**
				
Total cholesterol, mmol/L	4.74 ± 0.33	4.71 ± 0.29	4.68 ± 0.26	4.61 ± 0.27
Triglycerides, mmol/L	1.92 ± 0.26	2.26 ± 0.33^a^	1.75 ± 0.32	1.85 ± 0.24^b*^
Uric acid, μmol/L	314 ± 28	336 ± 33	323 ± 32	326 ± 32
Total polyphenols, mmol GAE/L	1.47 ± 0.14	1.61 ± 0.20	1.52 ± 0.15	1.46 ± 0.15
ORAC_(hydrophilic) ,_ mmol/L	1.41 ± 0.04	1.42 ± 0.05	1.38 ± 0.05	1.42 ± 0.06
ORAC_(lipophilic) ,_ mmol/L	0.74 ± 0.03	0.79 ± 0.03	0.73 ± 0.03	0.78 ± 0.03
FRAP, mmol Trolox equivalents/L	1.31 ± 0.10	1.12 ± 0.10^a^	1.42 ± 0.11	1.33 ± 0.12^b*^
Malondialdehyde, μmol/L	0.18 ± 0.02	0.14 ± 0.01	0.17 ± 0.01	0.15 ± 0.01
α-tocopherol, μmol/L	20.7 ± 2.28	19.6 ± 1.87	20.6 ± 2.47	19.0 ± 1.93
γ-tocopherol, mmol/L	2.29 ± 0.26^a^	2.35 ± 0.28	2.23 ± 0.21	2.85 ± 0.32^b*^

^1^Values are means ± SEM. Values in the same measure with different superscript letters a or b are significantly different (Student’s *t* test): *P < 0.05.

### Measures of antioxidant capacity and lipid oxidation

Table 3 compares the 5 hour response curves of biomarkers to test meals. The AUC_0-5h_ for hydrophilic ORAC was 7.5% (*P* = 0.008) higher and that of lipophilic ORAC 8.5% (*P* = 0.000) higher following the walnut meal, though total phenols and FRAP were unaffected by treatment. Neither the walnut nor the control meal influenced postprandial total cholesterol or uric acid responses. Although triacylglycerols increased similarly following both meals reaching concentrations of approximately 1.5 times baseline at 5 hours postmeal, the AUC_0-5h_ was higher following the walnut meal (*P* = 0.037). However, the walnut meal resulted in a lower postprandial MDA AUC_0-5h_ suggesting less lipid peroxidation. Ox-LDL AUC_0-5h_ did not differ between treatments but ox-LDL showed a significant decrease from baseline at 2 hours following walnut consumption.

**Table 3:** **Comparison of 5-hour area under the curve (AUC**_**0-5 h**_**) responses of biomarkers to test meals**^**1**^

**Biomarkers**	**Control (n = 16)**		**Walnut (n = 16)**		
	**LS Mean**	**95% CI**	**LS Mean**	**95% CI**	***P* value**
Total polyphenols, mmol GAE/L × h	6.96	5.87, 8.25	7.39	5.87, 9.39	0.076
Malondialdehyde, μmol/L × h	0.81	0.62, 1.01	0.75	0.63, 0.87	0.048
ORAC(hydrophilic), mmol/L × h	7.14	6.53, 7.76	7.68	7.10, 8.26	0.008
ORAC(lipophilic), mmol/L × h	3.80	3.43, 4.17	4.12	3.80, 4.45	0.000
FRAP, mmol Trolox equivalents/L	6.57	5.46, 7.69	6.47	5.37,7.58	0.920
Uric acid, μmol/L × h	1612	1317, 1869	1651	1317, 1943	0.591
Total cholesterol, , mmol/L × h	24.1	21.4, 26.6	23.8	21.1, 26.4	0.556
Triglycerides, , mmol/L × h	12.8	9.2, 17.2	14.5	10.6, 19.1	0.037
Oxidized LDL, mmol/L × h	-0.57	0.34, 0.97	-0.65	0.36, 1.16	0.910

^1^Values are least square means ± SEM; AUC_0-5h_ is the area under the concentration-time curve over 5 hours estimated by using the linear trapezoidal rule. A mixed model approach was used to compare AUC_0-5h_ among test meals adjusting for period effect and treating subjects nested within a sequence as random effects.

### Plasma catechins

Postprandial plasma was assayed for catechin, epicatechin, gallocatechin, epigallocatechin, epicatechin gallate (ECG), gallocatechin gallate (GCG), and epigallocatechin gallate (EGCG). Only ECG, GCG and EGCG were consistently detected in all plasma samples of participants tested. Total catechins is the sum of all catechins assayed that were detected. Table 4 shows increases in ECG, GCG and EGCG and in total catechins at 1 hour following the walnut meal.

**Table 4:** **Postprandial plasma catechin concentrations in response to test meals at baseline and at 1 and 2 hours following test meals**^**1**^

**Catechins**	**Control (n = 6)**		**Walnut (n = 6)**		
	**LS Mean**	**95% CI**	**LS Mean**	**95% CI**	***P* value**
Gallocatechin gallate GCG, ng/ml					
Baseline	0.40	0.08, 1.90	0.21	0.04, 0.99	0.99
1 hour	0.50	0.11, 2.39	4.63	0.97, 22.07	0.02
2 hour	0.41	0.07, 1.95	1.33	0.28, 6.32	0.40
Epicatechin gallate ECG, ng/ml					
Baseline	1.39	0.28, 6.85	1.21	0.24, 5.97	0.64
1 hour	0.25	0.05, 1.25	12.77	2.58, 63.14	0.01
2 hour	0.33	0.07, 1.65	6.18	1.25, 30.55	0.31
Epigallocatechin gallate, EGCG, ng/ml					
Baseline	0.93	0.15, 5.81	6.79	1.09, 42.24	0.99
1 hour	0.30	0.05, 1.89	108.60	17.45, 675.87	0.04
2 hour	1.72	0.28, 10.69	26.60	4.27, 165.55	0.15
Total catechins, ng/ml					
Baseline	9.06	1.84, 44.64	9.34	1.90, 45.99	0.99
1 hour	1.71	0.35, 8.43	132.95	26.99, 655.04	0.02
2 hour	4.71	0.96, 23.20	43.50	8.83, 214.33	0.87

^1^Values are least square means and 95% confidence intervals. A mixed model approach was used to compare concentrations between test meals adjusting for period effect and treating subjects nested within a sequence as random effects.

### Urine metabolites

Quantitative estimates of urinary phenolic metabolites excreted in urine collected in the first 12 hours (0–12 h) period and that collected in the 2nd (12–24 h) period following the test meals are shown in Table 5. Following the walnut meal, quantities of urolithin-A excreted were higher in both the 0–12 h and the 12–24 h urine.

**Table 5:** **Quantities of metabolites excreted in urine collected during 0 to 12 hours and during 12–24 hours following consumption of test meals**^**1**^

	**Control**		**Walnut**	
	**0–12 h urine**	**12-24 h urine**	**0-12 h urine**	**12-24 h urine**
3,4-Dihydroxyphenylacetic acid, mM	0.412 ± 0.353^a^	0.281 ± 0.182^b*^	0.375 ±197	0.401 ± 210
4-Hydroxyphenylacetic acid, mM	52.30 ± 35.68	49.73 ± 36.54	48.90 ± 25.37	72.44 ± 36.15
4-Methoxyphenylacetic acid, mM	6.45 ± 4.34	5.18 ± 2.86	4.59 ± 1.82^a^	7.71 ± 1.70^b*^
Urolithin A, μM	8.45 ± 0.88^a^	12.82 ± 28.03^a^	20.44 ± 32.18^b*^	100.59 ± 114.86^b*^

^1^All values are means ± SD. Values in the same measure with different superscript letters a or b are significantly different (Student’s *t* test): *P < 0.05.

## Discussion

This study was designed to examine the time-course effects of a walnut-rich test meal on plasma oxidative stress, antioxidant activity, concentration of tocopherols and phenolic molecules, and on the urinary excretion of phenolic metabolites. The postprandial state is characterized by lipemia along with increased oxidative stress and consequent inflammation and endothelial dysfunction [39]. We hypothesized that selected bioactive components in a walnut-rich meal are bioavailable and may help modulate postprandial oxidative responses. Our key findings were that compared to a refined fat meal, the walnut meal increased plasma γ-tocopherol, catechin monomers, hydrophilic and lipophilic ORAC and decreased concentrations of the lipid oxidation product MDA.

In this study, circulating triacylglycerols increased in reponse to the test meals whereas serum concentrations of total cholesterol did not change. Also, the 5-hour triacylglycerol response curve following the walnut meal was higher than that of the control meal. Although the total amount of fat in the test meals was equivalent, the refined meal contained a higher amount of saturated fat. It has been shown that the type of fat in a high fat meal influences postprandial triacylglycerols, with saturated fats producing less lipemia compared to unsaturated fat possibly due to their slower absorption rate [40]. Neverthless, the higher triacylglycerol concentration following the walnut meal was accompanied by decreases in plasma MDA indicating possible antioxidant protection due to walnut factors.

Results of studies on the antioxidant effects of nut-enriched diets have been mixed. A number of short-term randomized clinical trials showed reductions in lipid peroxidation measures especially MDA production associated with diets containing pecans [41], pistachios [42], almonds [43,44], and walnuts [45], whereas others showed no change in any of the measures of antioxidant status or lipid peroxidation [46,47]. On the other hand, studies that tested the postprandial effect of nuts consumed in the context of a meal usually showed antioxidant effects [23,27]. In addition, walnut meals were shown to acutely improve endothelial function [48] and reduce the postprandial inflammatory response in mononuclear cells in humans [24].

Of the assays used to determine changes in antioxidant activity of plasma, significant, albeit modest, increases in the AUC_0-5h_ for hydrophilic- and lipophilic-ORAC (7.5% and 8.5% respectively) were observed following walnut consumption, reflecting antioxidant activity in both aqueous and lipid plasma fractions. The ORAC assay measures hydrogen atom-donating capabilities of antioxidant molecules whose concentrations in plasma may have increased following the walnut meal. The AUC_0-5h_ of plasma polyphenols assayed by the Folin-Ciocalteau reagent was 6.5% higher after the walnut meal compared to the refined meal, but this result only approached statistical significance.

**Figure 1:**
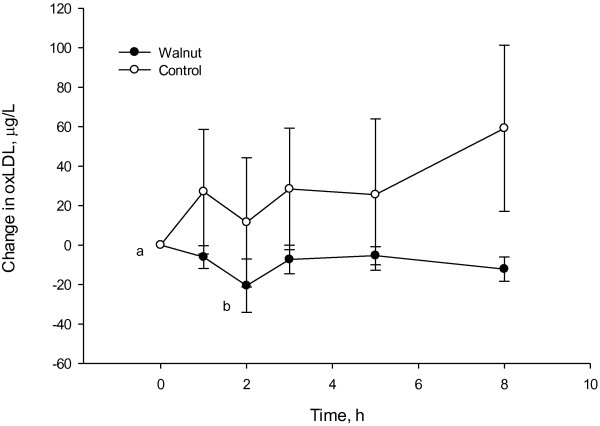
**Change in plasma oxidized LDL concentrations at baseline and after consumption of a walnut test meal compared to a control refined meal.** Values represent the men ± SEM of raw data. Oxidized LDL values were log transformed prior to analysis. *p < 0.05 versus baseline.

The oxidized LDL AUC_0-5h_ did not differ between diets, but following consumption of walnuts, oxidized LDL was reduced compared to baseline at 2 hours postmeal Figure 1. Oxidative modification of LDL is thought to play a role in the development of atherosclerosis and reductions in postprandial LDL have been reported following consumption of pecans [49] and pistachios [50].

When tested *in vitro*, walnut extracts show relatively high FRAP activity, highest among the nuts. We did not observe a consistent effect of a walnut meal on postprandial plasma FRAP concentrations. It has been suggested that the acute effect of foods such as fruits, berries, and wine on plasma FRAP is not due to their polyphenol content *per se* but may instead be explained by changes in the concentration of the endogenous metabolic antioxidant uric acid [51]. Blood urate concentrations increase following the consumption of fructose containing foods and in those circumstances, urate is the main contributer to FRAP of human plasma [28]. In the current study urate concentrations did not increase postprandially and did not differ between the two test meal interventions. These outcomes are in contrast to a recent study which resported increased postprandial FRAP following test meals of walnut oil and walnut skins in healthy overweight and obese adults [23].

**Figure 2:**
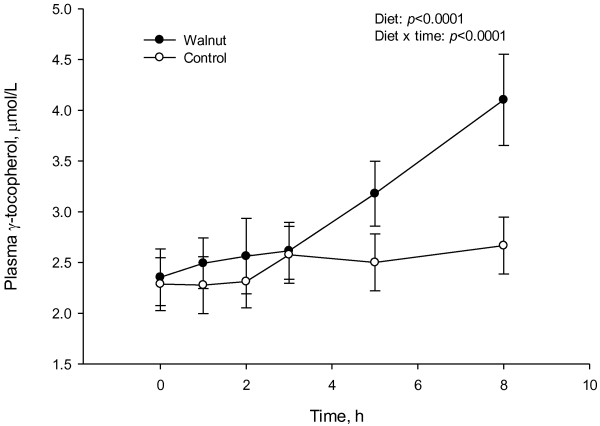
**Serum concentrations of plasma γ-tocopherol before and after a walnut and control test meal in a randomized crossover study design in healthy individuals.** Data are mean ± SE (n = 16).

There were no postprandial differences between interventions in plasma α-tocopherol concentrations, but γ-tocopherol nearly doubled at 8 h and remained higher at 24 h following the walnut meal Figure 2. *In vivo* studies report similar activities of α- and γ-tocopherol as chain-breaking antioxidants and inhibitors of superoxide generation, lipid peroxidation and LDL oxidation [52,53]. Previous studies have shown that γ-tocopherol is more potent in quenching reactive nitrogen species and decreasing protein nitration compared to α-tocopherol [54,55]. In a placebo controlled trial, supplementation with γ-tocopherol alone or combined with α-tocopherol resulted in marked decreases in measures of oxidative stress and inflammation in individuals with metabolic syndrome [56]. The acute increases in γ- tocopherol following the consumption of a γ-tocopherol rich food such as walnuts may be critical in attentuating the oxidant and inflammatory consequences of postprandial lipemia. It is worthwhile to note that the greatest increase in γ-tocopherol occurred at a later time point and therefore its contribution to antioxidant protection which occurred earlier in the study cannot be ascertained.

The walnut pellicle contains high levels of polymeric nonflavonoid hydrolyzable tannins (ellagitannins) [9], but small amounts of flavan-3-ol monomers or catechins. As mentioned previously, little data is published on the catechin composition of walnuts and that reported is variable and inconsistent (See the Additional file 1: Table S1). Studies in humans have shown bioavailability of catechins from tea or cocoa with absorption occuring in the small intestine [57-59]. In the current study, flavan-3-ol bioavailability was examined in 6 participants and although postprandial concentrations showed large inter-individual differences, all showed an increase in GCG, ECG, EGCG and in total catechins at 1 hour following walnut consumption. Due to the small number of subjects in this preliminary study and the fact that glucoronidase/sulfatase hydrolysis of plasma prior to analysis provides only indirect information about the nature of the catechins which appear in plasma, further study is needed on the bioavailability of catechins from walnuts.

Due to their bulkiness and high molecular weight, flavan-3-ol polymers are not absorbed through the gut barrier but are hydrolyzed *in vivo* to smaller aromatic acids and metabolites. In the current study, none of the phenylacetic acid derivatives tested in urine increased significantly following walnut consumption. However, walnuts are particularly rich in ellagitannins which are hydrolyzed *in vivo* to yield ellagic acid, a compound which is known for its low bioavailability [25,60]. Within the colon, ellagic acid undergoes microbial degradation to yield metabolites called urolithins characterized by a 6H-dibenzo[b, e]-pyran-6-1 nucleus. In the current study, the excretion of urolithin A increased following the walnut test meal especially in the 12–24 hour urine aliquots. As demonstrated previously [25,35,61], these microbial products are absorbed and appear in circulation as aglycones and their glucuronides. Additionally, when tested *in vitro*, these metabolites ameliorate TNF-α induced inflammation in human aortic endothelial cells, a mechanism which may reduce the risk of cardiovascular disease [62].

The strength of this study is its use of a crossover design for the intervention which minimizes confounders. Limitations include the fact that the participants were not blinded to the type of meal consumed. Also, the control meal, although refined, was not devoid of all possible dietary antioxidants. Due to the fact that both the background diet and the test meal have an effect on postprandial triglyceride responses, another limitation of the study is that only one day of standardized meals preceded the intervention. Also, not just the total fat but the fatty acid composition of the test meals must be standardized and similar as these influence postprandial responses [63]. Follow-up work may require larger participant numbers and more frequent sampling especially at earlier points after the test meal to capture small changes in components with short half lives. In addition, data on the urinary excretion of metabolites requires longer collection times, possibly as long as 48 hours following walnut consumption. Whether oxidative stress contributes to chronic disease is still a matter of debate. Lipemia promotes inflammation which has profound health consequences. Therefore, in addition to oxidative stress, future studies should assess the effect of incorporating walnuts in meals on postmeal concentrations of inflammatory mediators.

## Conclusions

In summary, our data indicate that a walnut meal compared to a refined high fat meal may modulate some of the oxidative effects of postprandial lipemia. Of the bioactives in walnuts, γ-tocopherol and possibly some catechins show postprandial increases which may influence markers of oxidation and antioxidant status. This study is not conclusive, but suggests a need to further explore the effect walnuts not only on lipemic stress but also on inflammation. The impact of these findings on long-term health remains to be elucidated.

## Competing interests

This study was partially supported by a grant from the California Walnut Commission, 1540 River Partk Drive, Suite #203, Sacramento, California 95815–4609. However, the Commission is not financing the publication of this manuscript. The authors have no conflicts of interest related to this study or its publication.

## Authors’ contribution

EH designed the study, performed the HPLC analyses and drafted the manucript. NT carried our the antioxidant assays and contributed to the manuscript. KO performed the statistical analysis. JS participated in designing the study. All authors read and approved the manuscipt.

## Supplementary Material

Additional file 1: Table S1 Summary of studies identifying major tannins and polyphenolic compounds in walnuts and of studies reporting *in vitro* antioxidant activity in walnut extracts. Click here for file
