# Supplementary material for: Effect of a walnut meal on postprandial oxidative stress and antioxidants in healthy individuals
Source: Nutr J. 2014 Jan 10;13:4. doi: 10.1186/1475-2891-13-4 (PMC3893411; doi:10.1186/1475-2891-13-4)
Supplement: Additional file 1: Table S1 — Summary of studies identifying major tannins and polyphenolic compounds in walnuts and of studies reporting in vitro antioxidant activity in walnut extracts. [file 1475-2891-13-4-S1.docx]

Additional file 1: Table S1

Summary of studies identifying major tannins and polyphenolic compounds in walnuts and of studies reporting *in vitro* antioxidant activity in walnut extracts

| Reference | Compounds identified | Amount |
| --- | --- | --- |
| ***Tannins and polyphenols*** | | |
| Daniel et al. (1989) (7) | Ellagic acid | 0.59 mg/g |
| Fukuda et al. (2003) (8) | Casuarictin  Glanserin A-C  Pedunculagin  Tellimagrandin I | 0.039 µg/g  16.4 µg/g  25.4 µg/g  58.6 µg/g |
| Gu et al (2004) (9) | Proanthocyanidin: Flavan-3-ol monomers | 69.3 µg/g |
| Colaric et al (2005) (10) | Phenolic acids (chlorogenic, caffeic, *p*-couramic, ferulic, sinapic, syringic, ellagic, juglone, syring-aldehyde) | 0.30 – 0.86 mg/g of phenolic acids |
| Li et al (2006) (11) | Ellagic acid  Valoneic acid dilactone | 0.29 mg/g  1.31 mg/g |
| Wu et al (2006) (12) | Total phenols | 15.56 ± 4.06 mg GAE/g |
| Kornsteiner et al (2006) (5) | Total phenols | 16.5 mg GAE/g |
| Venkatachalam et al (2006) (6) | Total tannins | 3.4±0.1 mg CE/g |
| Ito et al (2007) (13) | Glansreginin A  Catechin | 94.3 µg/g  7.3 µg/g |
| Gὀmez-Caravaca et al (2008) (14) | Catechin  Glansreginin A  Glansreginin B  Ellagic acid pentose dimer  Ellagic acid | 4.0 – 6.6 µg/g  76.3 – 335.6 µg/g  35.5 – 99.7 µg/g  33.0 – 37.2 µg/g  6.9 – 24.7 µg/g |
| Zhang (2009) (15) | Pyrogallol, p-hydroxybenzoic acid, vanillic acid, ethyl gallate, protocatechuic acid, gallic acid, pentahyroxydibenzolpyran |  |
| ***In vitro antioxidant activity of extracts*** | | |
| Wu (2004) (18) | Lipophilic ORAC  Hydrophilic ORAC | 4.84±1.25 µmol TE/g  130.57±35.2 µmol TE/g |
| Halvorsen (2006) (19) | FRAP | 13.126 ± 4.881 mmol/100 g |
| Bloomhoff (2006) (20) | FRAP with pellicle  Without pellicle | 23.073 mmol/100g  1.131 mmol/100 g |
| Pellegrini (2006)(21) | FRAP | 412.29 mmol Fe2+/kg |

CE catechin equivalents
